# Avoidance expression in rats as a function of signal-shock interval: strain and sex differences

**DOI:** 10.3389/fnbeh.2015.00168

**Published:** 2015-07-06

**Authors:** Richard J. Servatius, Pelin Avcu, Nora Ko, Xilu Jiao, Kevin D. Beck, Thomas R. Minor, Kevin C. H. Pang

**Affiliations:** ^1^Syracuse Veterans Affairs Medical Center, Stress and Motivated Behavior InstituteSyracuse, NY, USA; ^2^Department of Neuroscience, Stress and Motivated Behavior Institute, Rutgers Biomedical Health SciencesNewark, NJ, USA; ^3^Rutgers Biomedical Health Sciences, Graduate School of Biomedical SciencesNewark, NJ, USA; ^4^New Jersey Health Care SystemEast Orange, NJ, USA; ^5^Psychology, University of California at Los AngelesLos Angeles, CA, USA

**Keywords:** avoidance learning, motivation, anxiety disorders, diathesis-stress model, WKY, extinction learning, shock, temperament

## Abstract

Inbred Wistar Kyoto (WKY) rats express inhibited temperament, increased sensitivity to stress, and exaggerated expressions of avoidance. A long-standing observation for lever press escape/avoidance learning in rats is the duration of the warning signal (WS) determines whether avoidance is expressed over escape. Outbred female Sprague-Dawley (SD) rats trained with a 10-s WS efficiently escaped, but failed to exhibit avoidance; avoidance was exhibited to a high degree with WSs longer than 20-s. We examined this longstanding WS duration function and extended it to male SD and male and female WKY rats. A cross-over design with two WS durations (10 or 60 s) was employed. Rats were trained (20 trials/session) in four phases: acquisition (10 sessions), extinction (10 sessions), re-acquisition (8 sessions) and re-extinction (8 sessions). Consistent with the literature, female and male SD rats failed to express avoidance to an appreciable degree with a 10-s WS. When these rats were switched to a 60-s WS, performance levels in the initial session of training resembled the peak performance of rats trained with a 60-s WS. Therefore, the avoidance relationship was acquired, but not expressed at 10-s WS. Further, poor avoidance at 10-s does not adversely affect expression at 60-s. Failure to express avoidance with a 10-s WS likely reflects contrasting reinforcement value of avoidance, not a reduction in the amount of time available to respond or competing responses. In contrast, WKY rats exhibited robust avoidance with a 10-s WS, which was most apparent in female WKY rats. Exaggerated expression of avoidances by WKY rats, especially female rats, further confirms this inbred strain as a model of anxiety vulnerability.

## Introduction

Avoidance encompasses efforts, thoughts, and behaviors to forestall or eliminate a predicted aversive event or state. Abnormal or irrational expressions of avoidance are a core feature of anxiety disorders such as separation anxiety disorder, acute stress disorder and posttraumatic stress disorder (PTSD) (American Psychiatric Association, 2013). Neurobiological processes that increase the expression of avoidance or its resistance to extinction represent vulnerabilities to develop anxiety disorders, in keeping with diathesis models of anxiety disorders (Mineka and Zinbarg, 2006). Animal models of avoidance as a means to understand the etiology of anxiety disorders is consistent with recent organizational efforts in psychopathology for research domain criteria (RDoC) (Sanislow et al., 2010).

One animal model is discrete trial lever press or bar press avoidance. A lever press is not among species specific defense reactions that confound the interpretation of avoidance learning and expression (Bolles, 1970). In a signaled version, trials begin with a WS. A lever press during the WS prevents foot shock and constitutes an avoidance response. In the absence of an avoidance response, intermittent foot shocks are delivered for a period of time. A lever press after the initiation of foot shock terminates shock and initiated a safety period, which may be signaled. Lever presses during the safety period are not reinforced. As an arbitrary response, rates of nonspecific responding are generally low enhancing sensitivity (Servatius et al., 2008; Avcu et al., 2014). An escape response is generally acquired early in training with avoidance emerging and reaching asymptotic performance over several sessions of training (Servatius et al., 2008; Jiao et al., 2011, 2014; Pang et al., 2011; Avcu et al., 2014; Beck et al., 2014). Although avoidance is not expressed as quickly as other preparations (e.g., shuttle box), the slowness in acquisition is cited as a virtue (Bolles, 1970).

However, one area that is both basic and particularly troublesome is the apparent influence of WS durations on avoidance performance (Cole and Fantino, 1966; Jones and Swanson, 1966; Berger and Brush, 1975). Efficient avoidance is observed with WSs greater than 20 s. As the length of the WS decreases, the predominant behavior is escape; training with a fixed interval 10-s WS produced few avoidance responses, even fewer than a variable interval 10-s WS (Berger and Brush, 1975). The predominance of escape responding with WS durations shorter than 20 s may reflect an inability to *acquire* avoidance (resulting from response competition or failure to encounter the avoidance contingency to the degree necessary to support acquisition) or reduced *expression* of avoidance. The acquisition/expression issue is critical for interpretation and understanding neurosubstrates of avoidance. This anomaly was never pursued or elaborated, subsequent studies exploited the escape/avoidance patterns for understanding physiological concomitants (Brennan et al., 1992).

Beyond the basic science understanding of avoidance, strain differences in avoidance may be used to illustrate vulnerabilities. Among strains of rats exhibiting abnormally high degree of avoidance learning and expression, the WKY rat is unusual. Typically, rats selectively bred for rapid avoidance acquisition are less emotional and less stress reactive (Brush, 2003; Steimer and Driscoll, 2003). Further, rats selectively bred for emotionality exhibit an inverse relationship between emotionality and avoidance performance (Powell and North-Jones, 1974). However, the WKY strain is quintessentially stress-reactive (Paré, 1994), with an extensive literature linking the WKY rat as a model of depression (Paré, 1989; Carr et al., 2010). Yet, despite its behaviorally inhibited temperament (Servatius et al., 1998; Ferguson and Cada, 2004), the inbred WKY acquire lever press avoidance faster or to a higher degree than outbred Sprague-Dawley (SD) rats, especially female WKY rats (Servatius et al., 2008; Avcu et al., 2014). Previous work has exclusively examined avoidance acquisition with a 60 s WS. In that the relatively long CS provides ample opportunity for the WKY to overcome its inherent inhibited temperament, faster and greater expression of avoidance maybe an artifact of the CS duration chosen in initial studies as opposed to a generalized bias in avoidance acquisition and expression.

Therefore, the current study was conducted comparing acquisition of male and female SD and WKY rats with a 60 or 10-s WS. To determine whether behavioral patterns engendered during initial training interfere with learning once conditions are more conducive, a cross-over design was employed. After repeated sessions of extinction, rats were retrained with the WS duration not experienced during initial training. Thus, there were four phases: initial acquisition, initial extinction, re-acquisition, and re-extinction. For female SD rats, the experiment represents a replication and extension of research in the 1970s (Berger and Brush, 1975). We expected that male and female SD rats would not exhibit avoidances to an appreciable degree with a 10-s WS, this poor avoidance performance would transfer to subsequent training with a 60-s WS. For WKY rats, we expected the faster associativity of WKY rats (Ricart et al., 2011a,b) to offset the reduced exploratory time represented by the shorter WS period so that WKY rats still acquire and express avoidance to a higher degree than SD rats. The cross-over design would reveal whether poor avoidance performance would inhibit future avoidance (10–60 s cross) and to the extent generally high avoidance performance is affected by a shorter WS duration (60–10 s cross).

## Methods and materials

### Animals

Sprague-Dawley (SD) and Wistar-Kyoto (WKY) male and female rats (approximately 60–80 days of age at the start of the experiment) were obtained from Harlan Laboratories (Indianapolis, IN). Rats were housed in individual cages with free access to food and water in a room maintained on a 12:12 h day/night cycle for at least 2 weeks prior to experimentation. Experiments occurred between 0700 and 1900 h in the light portion of the cycle. All procedures received prior approved by the VA-NJHCS Institutional Animal Care and Use Committee in accordance with AAALAC standards.

### Group assignment

Naïve rats were tested for their acoustic startle response (ASR) as previously described (Servatius et al., 1998). A 15-min test session consisted of the presentation of 24 white noise bursts (100 ms with a 5-ms rise/fall time) at an intensity of 82, 92, or 102-dB, 8 trials of each sound level. The inter-stimulus interval varied between 15 and 25 s. Startle magnitudes at 102 dB white noise were used to match rats with strain and sex for random assignment to receive initial training with a 10 or 60-s WS. Therefore, the overall design was a 2 × 2 × 2 (Strain × Sex × WS duration) with 8 rats in each group. There were four phases of training: 10 acquisition sessions, 10 extinction sessions, 9 re-acquisition sessions, and 9 re-extinction sessions. Sessions occurred 3 times per week (every 2–3 days). A rat that failed to emit a lever-press response by the end of the fourth training session of initial acquisition was removed from the study. One male SD and one WKY rat in the 60-s group were dropped from the study for this reason (*N* = 7 for these two groups).

### Lever-press escape/avoidance training

The apparatus was described previously (Servatius et al., 2008). Training was conducted in 16 identical operant chambers (Coulbourn Instruments, Langhorne, PA). Each operant chamber was enclosed in a sound-attenuated box. Scrambled 2.0-mA foot-shock was delivered through the grid floor. The auditory WS was a 1000-Hz, 75-dB tone (10 dB above background noise). A 3-min intertrial interval (ITI) was explicitly signaled with a 5-Hz blinking cue light located above the lever. Graphic State Notation software (v. 3.02, Coulbourn Instruments, Langhorne, PA) controlled the stimuli and recorded response times.

Each session began with a 60-s stimulus-free period. A trial commenced with the presentation of the auditory WS. After either 10 s or 60 s, shocks (0.5-s, 1.0 mA) were delivered with a 3 s intershock interval until a lever press or 99 shocks were delivered. If a lever press occurred prior to the initiation of shock, the shocks were prevented, the WS terminated, and the safety period commenced; this event constitutes an avoidance response. If a lever press occurred after the initiation of shock, the shock train was immediately terminated, the WS ended and the safety period commenced; this event constituted an escape. During extinction training, both shock and the blinking cue light were deactivated. Each session consisted of 20 trials.

### Data analysis

All data are expressed as means ± the standard error of the mean. Statistical results are reported only where significant differences were found. For avoidance training, the number of avoidance responses and the number of shock received for each training session were compiled; shocks received only pertain to acquisition and reacquisition phases. Phases of the experimental were separately analyzed. *F*-tests for simple effects and Dunnett's and Dunn's tests were used for understanding contrasts.

## Results

### Acquisition

**Figure 1:**
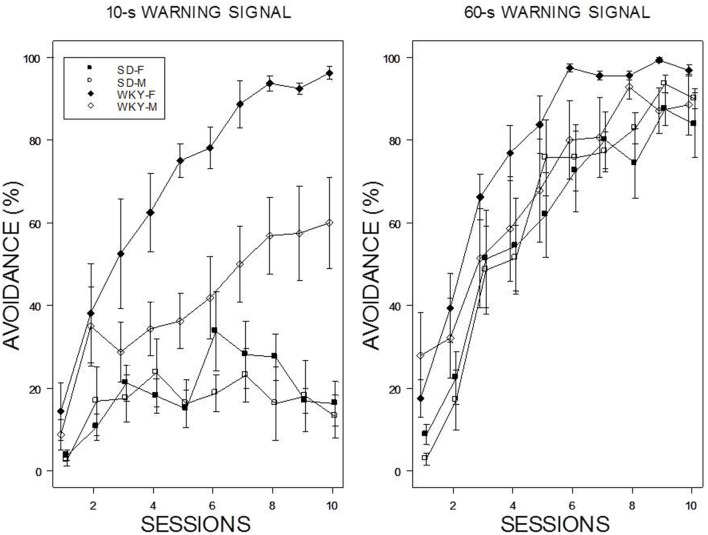
**Avoidance acquisition**. Avoidance performance using a 10-s WS (left panel) and 60-s WS (right panel). The legend is contained within the figure. Female WKY rats expressed avoidance to a higher degree than male WKY and SD rats. Male WKY rats achieved a modest level of acquisition with a 10-s WS, whereas SD rats generally did not express avoidance.

Acquisition with a 60-s WS progressed over the 10 training sessions with all rats attaining asymptotic levels of greater the 80% by the end of training (see Figure 1). In contrast, acquisition was poor with a 10-s WS in male and female SD rats; each exhibited less than 20% avoidance by the end of 10 session of training. In contrast, WKY rats generally acquired avoidance with the 10-s WS. Male WKY rats achieved a modest degree of avoidance (~60%) by the end of 10th session, whereas acquisition by female WKY rats with a 10-s WS was similar to that expressed with a 60-s WS. These impressions were confirmed with a 2 × 2 × 2 × 10 (Strain × Sex × WS × Sessions) mixed analysis of variance (ANOVA). Two triple interactions subordinate interactions and main effects: Strain × WS × Sessions, *F*_(9, 486)_ = 7.4 and Strain × Sex × Sessions, *F*_(9, 486)_ = 2.02, all *p*s < 0.05.

**Figure 2:**
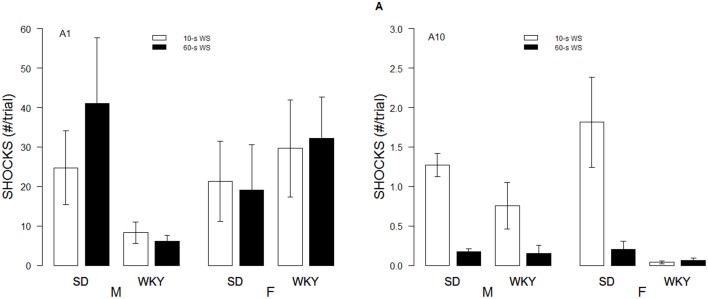
**Shocks experienced during acquisition**. Shocks rates per trial are depicted for the first session (A1, left panel) and 10th session of acquisition (A10; right panel) as a function of strain, sex, and WS duration. Initially, male WKY rats experienced less shocks. By the last session of acquisition, female WKY rats experienced the fewest shocks.

Although the WS duration affected the rates of avoidance acquisition, numbers of shocks received differed only as a function of Strain and Sex. These impressions were confirmed with a 2 × 2 × 2 × 10 (Strain × Sex × WS × Sessions) mixed-ANOVA. The triple interaction of Strain × Sex × Sessions, *F*_(9, 486)_ = 4.8, *p* < 0.05, superseded the subordinate interactions and main effects. Although all rats dramatically reduced the number of shocks received over training, male WKY rats only received roughly 60% of the shocks received by male and female SD and female WKY rats during the initial training sessions (See Figure 2).

### Extinction

**Figure 3:**
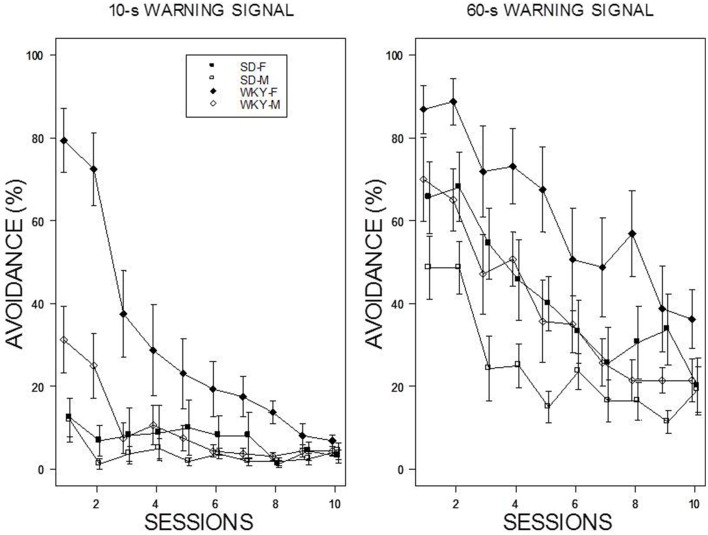
**Avoidance extinction**. Avoidance performance using a 10-s WS (left panel) and 60-s WS (right panel). The legend is contained within the figure. With respect to the 60-s WS, female rats exhibited slower extinction compared to males; WKY rats exhibited slower extinction compared to SD rats. In addition, the rate of extinction of WKY females was faster with a 10-s WS than 60-s WS.

For the 60-s WS, two dominant patterns were evident: WKY rats extinguished slower than SD rats, and female rats extinguished slower than male rats (see Figure 3). Thus, the slowest group to reduce avoidance responses was female WKY rats. For the 10-s WS, the relatively poor performance of all but the WKY female group precluded direct comparisons. All rats trained with a 10-s WS exhibited less than 20% avoidance responding by the end of the 10th session of extinction. These impressions were confirmed with a mixed ANOVA from which the four-way interaction was significant, *F*_(9, 486)_ = 2.64, *p* < 0.05. Of note, was the extinction patterns of the female WKY rats trained with either 10 or 60-s WS. Although avoidance performance in the last session of acquisition was similar, extinction with a 10-s WS was considerably faster than with a 60-s WS in female WKY rats.

### Reacquisition

**Figure 4:**
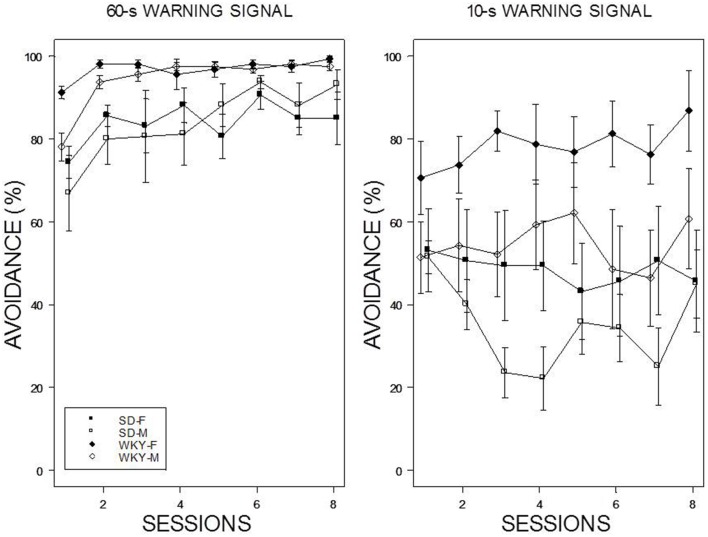
**Avoidance re-acquisition**. A cross-over design is depicted with rats trained with an initial 10-s WS, now trained with a 60-s WS (left panel), and rats trained initially with a 60-s WS, now trained with a 10-s WS (right panel). The legend is contained within the figure. A high degree of avoidance was expressed when rats initially trained with a 10-s WS were switched to 60-s WS, with slight increases over training. WKY rats expressed avoidance to a higher degree the SD rats. More modest levels of avoidance were expressed by rats initially trained with a 60-s WS but switched to a 10-s WS. Female WKY expressed avoidance to a higher degree than all other groups. Male SD rats decreased avoidance expression over sessions.

Reintroduction of the US and crossover to a new WS duration induced different patterns of avoidance responding in all groups except WKY females (see Figure 4). Switching from 10-s WS to the 60-s WS had an immediate impact on avoidance responding; avoidance responding was greater than 60% for all groups during the initial training session under the 60-s WS interval. There were little incremental performance differences over the remaining 9 sessions of training. Similar to initial acquisition with a 10-WS, female WKY rats performed better than all other groups at 60-s reaching nearly perfect asymptotic performance. Improved performance was evident in male WKY rats retrained with a 60-s WS, with asymptotic performance comparable to male WKY rats initially trained with a 60-s WS. The most dramatic differences were apparent in male and female SD rats, with each exhibiting avoidance performance levels in the initial session of reacquisition comparable to asymptotic performance of SD rats initially trained with 60-s WS, respectively. Switching from 60 to 10-s WS affected the avoidance performance of all groups, with only subtle changes the performance of female WKY rats. WKY males and SD females remained at modest levels of performance without improvement over training sessions. In contrast, SD males *reduced* their avoidance rates over sessions. Avoidance performance of SD males retrained with a 10-s WS dipped to the levels exhibited by rats initially trained with a 10-s WS. Overall, two general patterns were evident: WKY rats performed better then SD rats and females performed better than males. The mixed-ANOVA which yielded triple interactions of Sex × WS × Sessions, *F*_(7, 378)_ = 2.23, Strain × WS × Sessions, *F*_(7, 378)_ = 2.06, all *p*s < 0.05.

**Figure 5:**
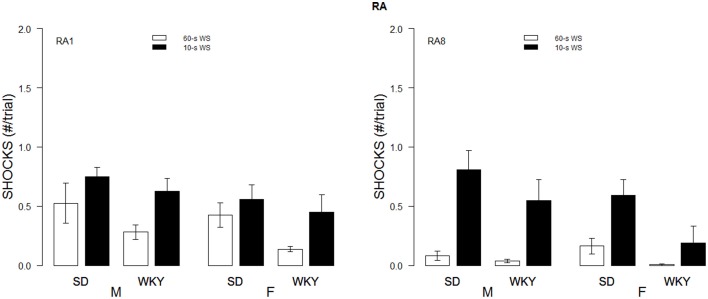
**Shock received during re-acquisition**. Shocks rates per trial are depicted for the first session (RA1, left panel) and 8th session of re-acquisition (RA8; right panel) as a function of strain, sex, and WS duration. Initial male WKY rats experienced less shocks. Cross over from 60 to 10 s led to increases in shock experienced (comparison of right panel of Figure 2 to left panel of this figure). Only female WKY rats reduced the number of shocks received from the 1st session of re-acquisition to the 8th session of re-acquisition.

As for the number of shocks received, those trained with the 10-s WS received more shocks than those with 60-s WS. Moreover, SD rats received more shock than WKY rats. These impressions were confirmed with a mixed-ANOVA which only yielded main effects of WS, *F*_(1, 54)_ = 4.41, and Strain, *F*_(1, 54)_ = 5.13, all p's < 0.05. Interestingly, SD rats who exhibited efficient escape responding during initial training with a 60-s WS—rarely experiencing more than a single shock on a given trial—were less efficient during reacquisition with a 10-s WS. SD rats retrained with a 10-s WS had numerous trials beyond the first trial of a session with more than one shock received whereas WKY rats retrained under similar conditions rarely experienced more than one shock on a given trial during reacquisition (see Figure 5).

### Re-extinction

**Figure 6:**
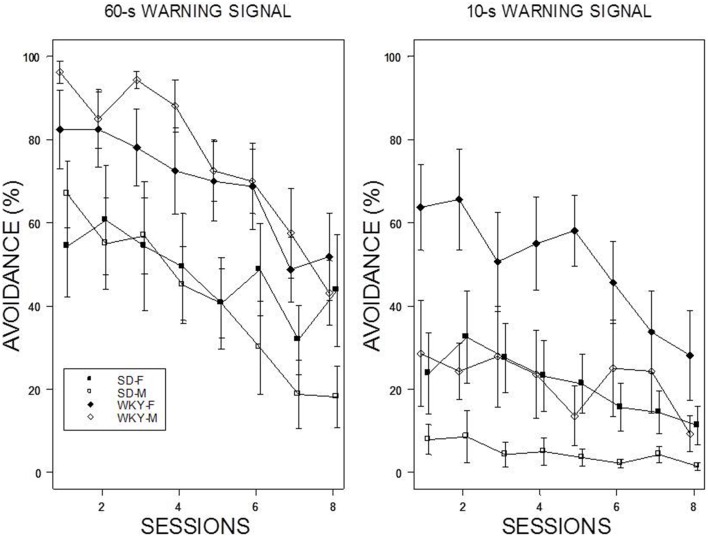
**Avoidance re-extinction**. A cross-over design is depicted of re-extinction with rats trained with an initial 10-s WS, now trained with a 60-s WS (left panel), and rats trained initially with a 60-s WS, now trained with a 10-s WS (right panel). The legend is contained within the figure. WKY rats exhibited slower re-extinction than SD rats with a re-acquisition was accomplished with a 60-s WS. Re-extinction after re-acquisition with a 10-s WS reflected levels of re-acquisition.

The dominant patterns exhibited during extinction were expressed during re-extinction, albeit with subtle differences (see Figure 6). Extinction after reacquisition with a 60-s WS showed clear strain differences with WKY rats exhibiting relatively slow extinction compared to SD rats. As with the initial extinction phase, the rates of extinction with the 10-s WS reflected asymptotic performance in the presence of the US. Thus, WKY females exhibited the slowest extinction; female SD and male WKY exhibited rapid rates from moderate levels of avoidance performance at the end of training. The mixed ANOVA revealed a triple interaction of Sex × WS × Sessions, *F*_(7, 378)_ = 4.75, which superseded the subordinate interactions and main effects, and a main effect of Strain, *F*_(1, 54)_ = 21.1, all *p*s < 0.05.

### Avoidance latency

**Figure 7:**
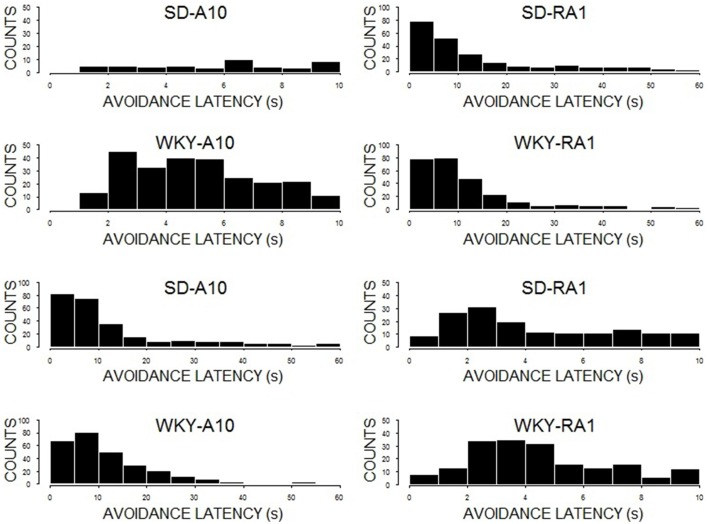
**Avoidance latencies**. Depicted are histograms of avoidance latencies for the 10th session of initial acquisition (A10; first column) and the 1st session of reacquisition (RA1; second column). The first row is SD rats initially trained with a 10-s WS; the total count of avoidances less than 10 s were 47 (A10) and 131 (RA1). The second row is SD rats initially trained with 60-s WS; the total count of avoidances less than 10 s were 158 (A10) and 157 (RA1). The third row is WKY rats trained initially with a 10-s WS; the total count of avoidances less than 10 s were 250 (A10) and 158 (RA1). The last row is WKY rats initially trained with 60-s WS; the total count of avoidances less than 10 s were 148 (A10) and 185 (RA1). Note the few response in by SD rats with training with a 10-s WS (first row), with the dramatically increase of avoidance responses lower than 10 s with reacquisition with a 60-s WS.

The impact of WS duration on latency to respond was cited as a potential explanation for performance differences between a 10 and 60-s WS. To facilitate interpretation of avoidance performance during training with 10 or 60-s warning, avoidance latencies were examined during the last session of acquisition (see Figure 7). Inasmuch as there were substantial differences between the two strains in avoidance performance in acquisition and reacquisition, we grouped rats by strain to ease presentation. Both SD (60%) and WKY (53%) rats exhibited disproportionately high rates of avoidance latencies less the 10 s during acquisition at 60 s (See Figure 5, bottom row). Moreover, the disproportionately high rate increased for SD (67%) and WKY (79%) rats during reacquisition with 60 s after initial training at 10 s. As for avoidance latency distributions for training with 10-s WS, the bulk of avoidance latencies were between 2 and 5 s (see Figure 5, top row).

## Discussion

In previous work it was observed that female SD rats failed to acquire avoidance with a 10-s WS (Berger and Brush, 1975). Consistent with this early work, female SD rats failed to appreciably acquire an avoidance response after 10 sessions of training with a fixed number of trials per session—considerably more extensive training than that previous. Moreover, male SD rats performed at a comparable level. Female and male SD rats did acquire efficient escape responses; both sexes received about one shock per trial during the last session of initial acquisition with a 10-s WS. The poor performance could be simply attributed to a lack of experience with the avoidance contingencies; however, all SD rats had experience with the avoidance contingency over the initial acquisition phase.

Failure to express avoidance was specific to the WS duration, in that after several sessions of extinction (absence of both shock and safety signal), avoidance acquisition was extremely rapid; avoidance rates during the last session of initial acquisition with a 10-s WS were nominal at 10–20%, but rose in the initial session of reacquisition with the introduction of a 60-s WS to 75% for female SD rats and 66% for male SD rats. The high avoidance response rates during reacquisition could have been attributable to expectancy to escape, that is, habitual responses that provided escape in 10 s WS, but now constituted avoidances with a 60-s WS. Response latencies during the last acquisition session with a 10-s WS for male (11.5 ± 0.3 s) and female (13.4 ± 3.2 s) SD rats, when both predominantly exhibited escape responses, reflect rapid responses with the onset of shock. In contrast, response latencies in the first session of reacquisition with a 60-s WS were considerably longer for both male (47.6 ± 6.0 s) and female (26.5 ± 2.0 s) SD rats. Failure to acquire or express avoidance with relatively short WS duration did not interfere with acquisition once conditions were more conducive. These data argue against the speculation that failure with 10-s WS is the result of proactive interference accruing from experience with unavoidable shock (Berger and Brush, 1975) similar to descriptions of interference in escape acquisition after experience with inescapable shock (Seligman, 1972). The high avoidance rates in the initial session of reacquisition by SD rats trained with a 10-s WS then switched to a 60-s WS argue strongly that SD rats acquired knowledge concerning the avoidance contingency, but did not express that knowledge through behavioral responses. The abrupt increase in avoidance responding from the marginal levels at 10-s WS to greater than 60% in the first session of re-acquisition with a 60-s WS resembles classic descriptions of latent learning (Tolman and Honzik, 1930).

The cross-over design also evaluated re-acquisition with a 10-s WS in those previously trained with a 60-s WS. Curiously, two patterns were then evident: (1) SD males decreased avoidance response rates over the next several sessions to near nominal levels, whereas response rates of SD females remained steady, but not incrementing with further exposure to the US during the reacquisition phase. This decline in male SD rats was evident even though avoidance response latencies of less than 10 s were virtually identical between the last session of acquisition with a 60-s WS and reacquisition with a 10-s WS (~50%). For female rats, no change in rates was evident between the last sessions of acquisition with a 60-s WS (~50%) and reacquisition with a 10-s WS (43–53% throughout reacquisition). Again, these data suggest that avoidance contingency may be acquired, but not expressed at a 10-s WS.

In the past, poor performance was suggested to be the result of: (a) reduced opportunity to respond, (b) schedule-induced differences in nonspecific responding, and (c) response interference (Berger and Brush, 1975). For example, the longer interval would presumably allow for greater opportunity to adventitiously encounter the lever and through trial and error. However, acquisition curves under varying WS duration find an abrupt increase in rates above 20 s WS with no advantage conferred by the greater opportunity. The supposition concerning indiscriminant responding—responding irrespective of the warning or safety signals was convincingly refuted by previous work, and fully supported here. The latter, response interference, may take different forms and will be more carefully addressed. One form of response interference postulates reflexive responses or response competition as the source of poor performance. Avoidance latencies may be used to address this point. In the simple case, an incompatible response would be evident early in the WS interval that dissipates as the interval lengthens (e.g., freezing). An alternative form with similarity would postulate that incompatible responses would be engendered more specifically with the shorter WS interval. In both, an incompatible response—whether associative or non-associative in nature—would interfere with the otherwise arbitrary lever press response and its acquisition. First, there is little empirical support that incompatible behaviors are differentially conditioned by 10 or 60-s. For example, a comparison of short and long WSs paired to a foot shock found similar degrees of freezing to the WS (Quinn et al., 2002; Barnet and Hunt, 2005). Given the assumption that an incompatible response such as freezing would be engender to the WS inhibiting either general exploration or specific responding, one would expect that avoidances in the 60-s group would have a considerably longer latency than 10 s. However, 60% of avoidance responses of SD rats trained with a 60-s WS occur with latencies less than 10 s. Thus, most of the avoidance responses of rats trained at 60 s have latencies within the window that would also be coincidental with incompatible freezing responses. Thus, response interference must be considered a highly unlikely explanation.

Typically, avoidance has a feed-forward impact on performance, that is, once the rat experiences the avoidance contingency avoidance responses tend to become more numerous to asymptotic performance generally exceeding 60%. Rats exceeding a plateau of 50% or better during a particular session go on to refine performance at better than 60% in subsequent sessions. However, this pattern—evident in those SD rats trained with a 60-s WS herein—was not evident in those trained with a 10-s WS. Of six SD rats that attained 50% or better in a particular session, not one exhibited asymptotic performance better than 60%. More dramatic was the decrease in avoidance performance over session in male SD rats initial training with 60-s WS then retrained with a 10-s WS. Together, these data suggest that for SD rats avoidance during 10-s WS was not as reinforcing as avoidance with 60-s signal duration.

One assumes that reinforcement of avoidance is the absence of foot shock, which is the same whether training with a 10 or 60-s WS. However, the perceived aversiveness of the foot shock may differ. Exposure to foot shock induces conditional and unconditional reductions in pain sensitivity (Fanselow and Sigmundi, 1986; Helmstetter and Fanselow, 1987). Conditional hypoalgesia is apparent through associations with discrete cues (Fanselow, 1986; Hagen and Green, 1988) and contextual elements (Matzel and Miller, 1987). Conditional hypoalgesia shows gradations in appearance relative to the duration of the cue (Seo et al., 2008). Consistent with these data, one may postulate that conditional hypoalgesia in the present studies is maximal more proximal to shock delivery. Accordingly, the differences between 10 and 60-s are postulated to reflect differences in motivation to avoid related to the imposition of conditioned analgesia; further studies are necessary to support this contention.

Two vulnerability factors for anxiety disorders were directly compared in their influence on avoidance learning and expression: temperament and sex. Consistent early work, females express avoidance to a higher degree than males (Van Oyen et al., 1981; Heinsbroek et al., 1983; Servatius et al., 2008; Avcu et al., 2014), although this sex difference was only evident in initial acquisition with 60-s WS and reacquisition with a 10-s WS. In addition, extinction was slower in female rats, particularly evident with initial extinction after training with 60-s WS.

A stronger factor is temperament, represented by the inbred WKY rat, which shows robust patterns of avoidance responding. With regard to acquisition with a 60-s WS, several patterns noted previously were evident here (although not stressed in the results section): (1) WKY rats lacked warm-up, as acquisition progressed WKY rats avoided on the first trial of a session, whereas SD rats generally exhibited an escape response (Servatius et al., 2008), (2) WKY rats exhibited slower rates of extinction (Servatius et al., 2008; Jiao et al., 2014). In stark contrast to SD rats, WKY rats acquired with a 10-s WS with females WKY rats expressing avoidance to a higher degree than male WKY rats. Although avoidance expression of male WKY rats initially trained with a 60-s WS was substantially faster than that with a 10-s WS, female WKY rats showed no differences between 10 and 60-s WS. Those similar learning curves allowed for the comparison of extinction; rates of extinction with 10-s WS were faster than that with a 60-s WS for female WKY rats. Regardless of WS duration WKY rats exhibited perseveration of avoidance for the first trial of an extinction session.

Unlike SD rats, WKY rats increased their expression of avoidance during the reacquisition phase with a 10-s WS. With the introduction of the 10-s WS period before initiation of shock, WKY rats not only matched the number of responses shorter than 10-s during initial training with the 60-s WS, but both male and female WKY rats increased the number of avoidance responses. For male WKY rats, the rates of avoidances with latencies shorter than 10 s during the last sessions of training with 60-s WS was 39% with avoidance rates in reacquisition with 10-s WS ranging from 48 to 60%. Similarly, female WKY rats initial acquisition rates were 64% increasing to 70–86% during training with 10-s WS. These data suggest that WKY rats are more flexible in modifying established avoidance responses.

For WKY rats, it is not just the degrees of avoidance expression but the manner in which avoidance is expressed. Once acquired, WKY rats begin each session with avoidance (lack of warm up). Early avoidance is a double-edged sword. Fewer foots shocks are experienced by the rat. However, the rat is insensitive to environmental changes. This insensitivity is clearly evident if there is continued immediate contingent feedback either in the form of WS termination or initiation of safety signal. Under such conditions, avoidance expression continues during extinction (disconnection of the US) without appreciable decline for at least 8 sessions (Servatius et al., 2008). If contingent feedback is discontinued during extinction (as was done herein), avoidance rates gradually decline to nominal levels. Whereas a number of SD rats (male and female) extinguish to the degree that entire sessions lapse without a single lever press, almost all WKY rats continue to respond on the first trial of a session, even when that is the only response for the entire session. These early session responses, highly specific to the presence of the WS, continue even though the environmental conditions over the training session are essentially the same. Such lever presses to the WS in extended absence of foot shock are reminiscent of excessive worry.

Worry is a core feature of generalized anxiety disorder. Inhibited temperament is not only strongly associated with social anxiety (Hudson et al., 2011) and general anxiety disorder (Moffitt et al., 2007; Hudson et al., 2011), but obsessive compulsive disorder (Ivarsson and Winge-Westholm, 2004) and posttraumatic stress disorder (Myers et al., 2012a,b); these anxiety disorders are more prevalent in females (Pigott, 2003; Steel et al., 2014). Female inbred WKY rats are a homogenous group to understand neurobiological influences on avoidance and expressions of avoidance in the development of anxiety disorders. Perseveration of early session avoidance responses during extinction could provide a specific target for therapeutics aimed at reducing worry.

## Summary

Extending long standing work, the avoidance performance of SD rats trained with a 10-s WS was poorer than when training was accomplished with a 60-s WS. The cross-over design illuminated poorer performance as *expression* not *acquisition* of avoidance. Reduced avoidance expression in SD rats likely reflects reduced reinforcement value with a 10-s WS. As models of inhibited temperament, inbred WKY rats (especially female WKY rats) expressed avoidance to a greater degree than outbred SD rats regardless of WS duration.

### Conflict of interest statement

The authors declare that the research was conducted in the absence of any commercial or financial relationships that could be construed as a potential conflict of interest.
